# Effects of Açai (*Euterpe oleracea *Mart.) berry preparation on metabolic parameters in a healthy overweight population: A pilot study

**DOI:** 10.1186/1475-2891-10-45

**Published:** 2011-05-12

**Authors:** Jay K Udani, Betsy B Singh, Vijay J Singh, Marilyn L Barrett

**Affiliations:** 1 Medicus Research LLC, Northridge, CA 91325, USA; 2 UCLA School of Medicine, Department of Medicine, Los Angeles, CA 90024, USA; 3 Pharmacognosy Consulting, Mill Valley, CA 94941, USA

## Abstract

**Background:**

The purpose of this study was to evaluate the effect of açai fruit pulp on risk factors for metabolic disorders in overweight subjects. The açaí palm (*Euterpe oleracea *Mart.), which is native to South America, produces a small, black-purple fruit which is edible. The fruit has recently become popular as a functional food due to its antioxidant potential. Although several studies have been conducted in vitro and with animals, little is known about the potential health benefits in humans aside from an increase in plasma anti-oxidant capacity. Metabolic syndrome is a condition which is defined by a cluster of risk factors for cardiovascular disease and/or type-2 diabetes. Preliminary studies indicate that a reduction in reactive oxygen species can assist in the normalization of the metabolic pathways involved in this syndrome.

**Methods:**

This was an open label pilot study conducted with 10 overweight adults (BMI ≥ 25 kg/m^2 ^and ≤ 30 kg/m^2^) who took 100 g açai pulp twice daily for 1 month. The study endpoints included levels of fasting plasma glucose, insulin, cholesterol, triglycerides, exhaled (breath) nitric oxide metabolites (eNO) and plasma levels of high sensitivity C-reactive protein (hs-CRP). The response of blood glucose, blood pressure and eNO to a standardized meal was determined at baseline and following the 30 day treatment.

**Results:**

Compared to baseline, there were reductions in fasting glucose and insulin levels following the 30 day treatment (both p < 0.02). There was also a reduction in total cholesterol (p = 0.03), as well as borderline significant reductions in LDL-cholesterol and the ratio of total cholesterol to HDL-cholesterol (both p = 0.051). Compared to baseline, treatment with açai ameliorated the post-prandial increase in plasma glucose following the standardized meal, measured as the area under the curve (p = 0.047). There was no effect on blood pressure, hs-CRP or eNO.

**Conclusion:**

In this uncontrolled pilot study, consumption of açai fruit pulp reduced levels of selected markers of metabolic disease risk in overweight adults, indicating that further studies are warranted.

## Background

People who are overweight, particularly those with excess central adipose tissue, are at risk of developing dysfunctions in lipid and glucose metabolism resulting in a propensity for cardiovascular disease and/or type-2 diabetes. This cluster of symptoms, which includes elevated blood pressure, elevated fasting blood glucose levels, elevated triglycerides and reduced levels of high-density lipoprotein cholesterol, has been defined as metabolic syndrome[1].

Oxidative stress, which is an imbalance between the generation of free radical species and the activity of anti-oxidant defense mechanisms, is thought to be one of the underlying mechanisms behind the risk of cardiovascular disease and diabetes associated with obesity[2]. Subjects who are obese are more likely to have higher levels of oxidative stress than those of normal weight. In addition, weight loss is associated with a decrease in oxidative stress[3]. Further, analysis of endogenous anti-oxidant protection in subjects with metabolic syndrome indicate that it is depressed[4]. And, subjects with metabolic syndrome also tend to display an increase in oxidative damage, measured as elevated lipid peroxidation and elevated protein carbonyls[3]. Oxidative stress has also been shown to enhance insulin resistance and it has been suggested that antioxidant therapy may reduce insulin resistance in diabetic patients[4,5].

Diets which are high in fruits and vegetables, are reported to increase plasma antioxidant capacity[6]. In addition to vitamins C, E and beta-carotene, fruits and vegetables contain phenolic compounds that contribute to their antioxidant capacity[7]. The inverse relationship between fruit and vegetable intake and the risk of cardiovascular diseases and diabetes has been associated with antioxidant capacity of these foods and in their phenolic content[8,9]. Preclinical studies have reported that polyphenolics compounds have beneficial effects on glucose absorption, insulin levels and lipid metabolism[10].

The fruit of the açaí palm (*Euterpe oleracea *Mart.), which is native to South America has recently become popular as a functional food due to its antioxidant potential[11]. The edible fruit is round, black-purple in color, about 1-inch (25 mm) in diameter and contains a single large seed. Macerating the pulp of the fruit produces a viscous liquid which is approximately 2.4% protein and 5.9% lipid (by weight)[12]. Analysis of fatty acid composition reveals that monounsaturated oleic acid is the primary fat, present at 56.2%, followed by palmitic (saturated fatty acid; 24.1%) and linoleic acid (polyunsaturated; 12.5%)[11]. Analysis of phenolics in the fruit revealed the presence of anthocyanin 3-glycosides, ferulic acid, epicatechin, p-hydroxy benzoic acid, gallic acid, protocatechuic acid, catechin, ellagic acid, vanillic acid, p-coumaric acid and gallotannins[12]. Cyanidin-3-monosaccharides are reported to be present in the fresh pulp at a concentration of 1040 ± 58 mg/L[13]. More specifically, the glucosides cyanidin-3-rutinoside and cyanidin-3-glucose, were measured at concentrations of 1.93 and 1.17 mg/g dry weight, respectively, with a total anthocyanin content of 3.19 mg/g dry weight[11]. Açai pulp is reported to have an antioxidant capacity of 48.6 μmol Trolox equivalents (TE)/ml as measured using the oxygen radical absorbance capacity (ORAC) assay[12]. Anthocyanins are generally considered to be the major contributors to the antioxidant activity of the pulp. However this concept has been challenged by a group of researchers who estimated the contribution of the anthocyanins to be just 10% of the total in vitro antioxidant capacity and suggested that other, unidentified, antioxidant constituents exist in the fruit[14]. This group of researchers compared the Total Oxidant Scavenging Capacity (TOSC) values for cyanidin-3-glucoside and cyanidin-3-rutoside to their corresponding concentrations in açaí preparations and found that the açaí preparations had significantly higher TOSC values than the calculated capacity due to constituent cyanidin glycosides.

In addition to the in vitro assays, the antioxidant potential of preparations of açaí pulp and juice has also been reported in animal and human studies. Experiments with rats revealed that a diet supplemented with 2% açai pulp (dry wt/wt) for 6 weeks caused a reduction in protein oxidation compared to control animals. Protein oxidation was measured as a decrease in carbonyl protein and an increase in protein sulfhydryl groups. There were also beneficial effects, compared to controls, on antioxidant enzyme activity, measured as an increase in serum paraoxonase, which is associated with prevention/inhibition of lipoprotein oxidation. In animals fed a hypercholesterolemic diet with or without açai pulp, there was a decrease in serum superoxide dismutase (SOD) activity compared to controls. SOD is an enzyme which is induced in response to oxidative stress and converts superoxide radicals into hydrogen peroxide[15]. In humans, plasma anti-oxidant capacity (measured using the ORAC assay) in humans increased up to 3 fold with a single dose of 7 ml açaí pulp/kg body weight compared to the control beverage (p < 0.01), with a *t*-max of 3 hours[16]. The maximum plasma concentration (C_max_) of total anthocyanins, measured as cyanidin-3-glucoside was reached 2.2 hours after consumption of the pulp.This pharmacokinetic study included healthy volunteers, who were dosed after a 72 hour dietary washout phase and an overnight fast. The subjects consumed a diet low in antioxidants that excluded the majority of dietary polyphenolics. The antioxidant capacity was measured as the ratio of TE for each time point compared to baseline, divided by the dose volume administered to the subject.

As previously stated, reducing the production of reactive oxygen species is hypothesized to assist in the normalization of metabolic pathways that lead to the onset of diabetes, endothelial dysfunction and cardiovascular disease[2]. In a test of that hypothesis, this pilot study was designed to evaluate the effects of a proprietary preparation of açai pulp in overweight subjects who are at risk for developing metabolic syndrome. Several studies have been conducted on the effects on açai preparations in vitro and in animals, but little is known about its effects on humans. Epidemiological and experimental studies point to the potential benefits of antioxidants in ameliorating the risks of cardiovascular disease and diabetes type II, however the results from clinical studies with antioxidant vitamins have been equivocal. The study measured endpoints before and after administration of açai pulp for one month. Measurements included levels of fasting plasma glucose, insulin, cholesterol and triglycerides. Exhaled (breath) nitric oxide metabolites (eNO) and plasma levels of high sensitivity C-reactive protein (hs-CRP) were measured as indicators of inflammation. The response of blood glucose, blood pressure and eNO to a standardized meal was also determined.

## Materials and methods

### Investigational Product

The preparation used in this study was Sambazon^® ^Açai Smoothie Pack (Sambazon Inc, San Clemente, CA), a frozen product containing a puree of açai (*Euterpe oleracea *Mart.) pulp that is designed to be made into a smoothie. The açai pulp was pasteurized and manufactured in a GMP facility in Brazil. Initially a bulk pulp containing 14% dry açai solids was produced and this was diluted with water to produce the Smoothie Pack which contained 11% solids (11 g). The bulk pulp contained 6.42 g fatty acids per 100 g: 61.4% octadecenoic acids (18:1; including oleic acid), 20.8% hexadecanoic acids (16:0; palmitic) and 11.2% octadecadienoic acids (18:2; linoleic acid). The bulk pulp also contained 3.5 mg/ml total phenolics measured as gallic acid equivalents and 0.77 mg/ml total anthocyanins measured as cyanidin-3-glucoside equivalents (as analyzed by Brunswick Laboratories, MA). In vitro antioxidant capacity of the hydrophilic contents, measured as micromole Trolox equivalency using the ORAC assay, was 46 μmole TE/ml[17]. This activity accounted for 98.9% of the ORAC activity; only a little more than 1% of the in vitro antioxidant capacity was found in the lipid-soluble fraction. Nutrition analysis of the 100 g Pack performed by Silliker, Inc. Southern CA Laboratory (Cypress, CA) revealed the 100 g Pack contained 71.8 calories, 5.8 g total carbohydrate (<0.25 g sugars), 4.9 g total fat (1.1 g saturated fat), 5.33 g fiber and 1.0 g protein.

The participants were instructed to place the contents of the Sambazon^® ^Açai Pulp into a Blender Bottle^®^, adding water and up to 4 g (1 packet) of sugar as desired making a "smoothie". As a measurement of compliance, the participants took a photo of each smoothie and e-mailed the photo to the research coordinators before drinking. The dose was 100 g twice daily, taken in the morning and evening. A diary was kept noting whether each smoothie was consumed and the corresponding photo sent.

### Subjects

Subjects were included if they were 18-65 years of age, had a BMI ≥ 25 kg/m^2 ^and ≤ 30 kg/m^2 ^and agreed to all study visits and procedures as well as not to initiate/change any exercise or diet programs during the study. Females of child-bearing age agreed to use approved forms of birth control. Subjects were required to have a cell phone with a camera along with the ability to transmit photos and to agree to use their cell phone for study purposes. Subjects were excluded if they had any major systemic, inflammatory or chronic diseases, had an infection, used diabetic medications in the 4 weeks prior to the study, used insulin currently or in the past 3 years, had symptomatic hypoglycemia in the past month, used immunosuppressive drugs in the prior 5 years, smoked cigarettes, or abused alcohol/drugs. Females who were pregnant or lactating were also excluded. Subjects with a baseline exhaled nitric oxide (eNO) measurement of > 35 ppb were also excluded. Intake of steroids, anti-inflammatory drugs, multi-vitamins and anti-oxidants were prohibited during the study.

### Study Design

This was an open label pilot study conducted with 10 overweight adult men and women who took Sambazon^® ^Açai pulp 100 g twice daily for 1 month. As this was a pilot study and there was no prior human data on the effects of the Açai product from which to perform a power calculation, the sample size was set at 10. The study was conducted at the Staywell Research clinical research site located in Northridge, CA. and was monitored by the Medicus Research Contract Research Organization. Institutional Review Board approval was obtained from the Copernicus Group IRB (Cary, NC) prior to the initiation of any study related procedures. Good Clinical Practices (GCP) were followed throughout the study and all subjects gave informed consent according to GCP guidelines.

The clinical study began in October 2009 (first subject in) and lasted until December 2009 (last subject completed). The subjects in the study came to the research clinic for a total of 4 visits (V1-V4). The maximum treatment duration was 4 weeks. There was a window of ± 2 days for V2, V3 and V4. The subjects were given a handout listing foods containing nitrates (for example bacon and hot dogs) and asked to avoid these foods for the duration of the study.

V1 was a screening visit during which the subjects were screened for inclusion and exclusion criteria, gave informed consent and medical history, received a physical examination, had a review of current medications, demographic assessment, urine collection (pregnancy test for females of child bearing age) and were dispensed standardized frozen foods which were the only foods to be consumed during the 24 hours prior to the next visit. V2 was the baseline visit for which the subjects arrived fasting (since mid-night the night before), having avoided exercise and alcohol for 24 hours and caffeine since 8 pm the night before. They had a physical exam during which vital signs were noted along with anthropometric measures (body weight, height and circumferences of waist/hip). Baseline determinations were made of blood glucose (capillary), insulin, cholesterol (total cholesterol, HDL-cholesterol, LDL-cholesterol and triglycerides), exhaled (breath) nitric oxide metabolites (eNO) and high sensitivity C-reactive protein (hs-CRP). Blood glucose was measured using finger prick samples (Bayer Ascensia Contour Glucometer). Insulin was monitored using a chemilumiscent method on a DPC Immulyte Analyzer. Lipid levels and hs-CRP were determined using an Olympus AU 2700 Autoanalyzer. eNO was measured using a devise developed by Insight eNO System (previously Apieron Inc, Menlo Park, CA, now Aerocrine AB, Sweden). The devise measures eNO via an optical biosensor that undergoes a change in its optical absorbance when binding with nitric oxide[18].

Following the visit's baseline determinations, the subjects consumed a standardized meal (57 g carbohydrate, 50 g fat, 18 g protein, nitrate-free; Stouffer's Fettuccini Alfredo, Nestlé USA, Wilkes-Barre, PA). eNO was determined 1 hour and 2 hours after the meal. Blood pressure and blood glucose (capillary) levels were determined 30, 60, 90 and 120 minutes after the meal. After all tests were complete, the study products were dispensed as well as diaries (Daily dosing diary and 3-day food recall diary). V3 was scheduled 2 weeks following visit 2. During this visit, compliance was assessed by collecting product packaging dispensed at the previous visit. The diaries administered at the last visit were collected and new diaries were dispensed. Also dispensed were standardized frozen foods to be consumed during the 24 hours prior to next visit (V4). During V4 the measurements conducted during V2 were repeated. Compliance was again assessed and diaries administered at the last visit were collected.

### Statistical Analysis

Excel 2003 (Microsoft Corp, Redmond WA) was used for data entry, validation, restructuring, calculating changes in variables over time, reorganizing and reformatting results, and preparing the graph. Statistical analyses (descriptive statistics and Student t tests) were performed using SPSS Base System ver. 17 (IBM SPSS Inc., Chicago IL.).

The percent changes in capillary blood glucose levels from time zero to 120 minutes after the meal were determined for each individual. The averages of the individual percent changes were calculated and plotted as minutes from time zero (baseline). The area under the curve (AUC) was calculated using the Trapezoidal Rule.

## Results

The demographics of the ten participants in the study is given in Table 1. Their body measurements did not change significantly over the month of the study. Three-day food recall diaries were collected at baseline and after 4 weeks of treatment. Based on the assessment of macronutrient content (fat, protein, carbohydrates) and total caloric intake, the diet of the subjects did not change over the course of the study. The subjects took 100 g açai pulp twice daily for one month. Compliance, as measured using photographs of the prepared smoothies, was 100%.

**Table 1:** Participant Demographics

Number	10
**Age (years)**	18-46 (mean 28.1)

**Sex**	5 men, 5 women

**Race**	9 Hispanic, 1 Caucasian

**Marital status**	5 single, 4 married, 1 divorced

**Body weight (lbs)**	173.0 ± 18.1*

**BMI**	27.4 ± 1.8*

**Waist/hip ratio**	0.86 ± 0.06*

* mean ± standard deviation

The subjects mean baseline fasting glucose level was 98.0 ± 10.1 mg/dl, which is on the upper edge of normal (70 to 99 mg/dl) (Table 2)[1]. After one month administration of the açai pulp, the mean fasting glucose level decreased significantly to 92.8 ± 10.9 mg/dl (p = 0.018). The mean plasma fasting insulin levels for the group decreased from 8.9 ± 54 μU/ml at baseline, to 6.7 ± 33 μU/ml (p = 0.017). (Table 2)

**Table 2:** Measurements before and after administration of açai pulp

Measurement	Time	Mean	Std. Deviation	**Sig**.
**Fasting Glucose (mg/dl)**	Baseline	98.0	10.1	.018
		
	Day 30	92.8	10.9	

**Insulin (μU/ml)**	Baseline	8.92	5.4	.017
		
	Day 30	6.68	3.3	

**Serum Cholesterol (mg/dl)**	Baseline	159.2	37.4	.030
		
	Day 30	141.8	28.3	

**Cholesterol Ratio**	Baseline	3.79	1.0	.051
		
	Day 30	3.42	0.9	

**VLDL (mg/dl)**	Baseline	26.2	13.5	.111
		
	Day 30	20.9	8.3	

**LDL (mg/dl)**	Baseline	90.1	29.1	.051
		
	Day 30	78.1	25.3	

**HDL (mg/dl)**	Baseline	42.9	8.4	.953
		
	Day 30	42.8	9.3	

**Triglycerides (mg/dl)**	Baseline	130.8	67.3	.116
		
	Day 30	104.2	41.6	

Measurements of glucose, insulin and lipid levels before and after administration of Sambazon açai for 30 days (given as means and standard deviations) were compared using paired t-tests to determine the significance of the change from baseline.

Consumption of açai reduced total cholesterol from 159 ± 37 mg/dl to 142 ± 28 mg/dl (p = 0.03). Consumption of açai also reduced the levels of low density lipoprotein (LDL)-cholesterol from slightly elevated to within the optimal range (p = 0.51). Levels of high density lipoprotein (HDL)-cholesterol did not change significantly following intake of açai. The ratio of total cholesterol to HDL-cholesterol, obtained by dividing the HDL cholesterol level into the total cholesterol, was reduced from 3.79 ± 1.0 at baseline to 3.42 ± 0.9 after treatment (p = 0.051). There was no significant change over time in levels of very low density lipoprotein (VLDL) cholesterol or triglycerides (Table 2).

The mean baseline measurement for highly sensitive C-reactive protein (hs-CRP) was 2.7 ± 3.0 mg/L and did not change significantly following administration of açai.

**Figure 1:**
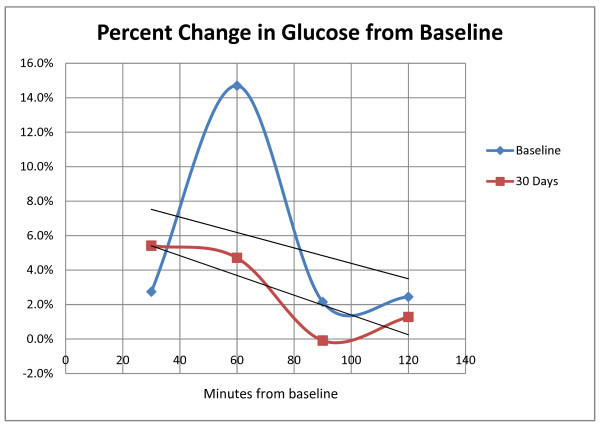
**Post-prandial levels of plasma glucose were measured before and up to 120 minutes after consumption of a standardized meal**. The determination of the percent change in glucose from baseline was made at the start of the study (baseline curve) and after administration of Sambuzon açai for 30 days (30-day curve). The trend lines for the baseline and 30 day measurements are depicted as two straight lines.

The effects of consumption of a standardized meal (57 g carbohydrate, 50 g fat, 18 g protein, nitrate-free) on plasma glucose, mean exhaled nitric oxide (eNO) and blood pressure were determined before (baseline) and after 30 days of consumption of açai pulp. The averages of individual percent changes in capillary blood glucose levels from time zero to 120 minutes after the meal are plotted in Figure 1. At the baseline visit, the postprandial glucose levels peaked at 60 minutes, with an increase of 14.7%. After 30 days of consumption of açaí, the percent change from baseline at 60 minutes was only 4.7%. The glucose area under the curve (AUC) measurement for the baseline visit was 205.6 ± 18.6 and after 30 days of açaí was 189.7 ± 26.3 (mean ± standard deviation). The difference was significant (p = 0.047). Thus, consumption of açai pulp for 30 days significantly reduced the post-prandial increases in glucose levels following the standardized meal. There were no significant changes in blood pressure or in eNO. Mean baseline blood pressures were 119 ± 7 mmHg systolic and 72 ± 9 mmHg diastolic at baseline. The mean exhaled nitric oxide (eNO) level at baseline was 17.5 ± 7.6 ppb, within the normal range of less than 25 ppb.

Açai pulp was safely consumed. There were no adverse events or changes in vital signs (body temperature, pulse or respiratory rate).

## Discussion

According to the American Heart Association[19], a BMI of 25 kg/m^2 ^corresponds to about 10 percent over ideal body weight. People with BMIs in this range are considered to have an increased risk of developing dysfunctions in glucose/insulin levels and lipid metabolism. The subjects in this study had a mean BMI of 27.4 and were categorized as overweight (BMI 25 to 29.9). Consumption of Sambazon^® ^Açai by this population, reduced plasma levels of fasting glucose, insulin and total cholesterol. Consumption of Sambazon^® ^Açai also reduced the post-prandial increase in plasma glucose following a meal.

Epidemiologic studies on the prevention of diabetes have shown that overweight subjects randomized to various lifestyle interventions who demonstrated reductions in fasting glucose of 4 mg/dL (3.6%) and in cholesterol of 5 mg/dL (2.3%) had a significant reduction (58%) in the risk of becoming diabetic[20]. In this study, the reductions in fasting glucose and total cholesterol compared to baseline were 5.3% and 10.6%. These reductions are greater than those deemed necessary for a change in risk status.

The potential effects of other anthocyanin-rich preparations on glucose levels in subjects with ether diabetes type 2 or metabolic syndrome have been tested clinically. Choke berry (*Aronia melanocarpa*) juice (200 mls) given to subjects with type 2 diabetes (200 ml/day for 3 months) reduced fasting glucose levels (13.3 ± 4.5 to 9.1 ± 3.0 mmol/l) and hemoglobin (Hb)A1c levels (9.3 ± 2.2 to 7.5 ± 1.3) compared to baseline (both p < 0.001)[21]. However, another study in which an extract of choke berry (3 × 100 mg/day) was given to 25 subjects with metabolic syndrome for 2 months, did not show any effect on fasting plasma glucose levels (baseline 92.9 ± 11.0 mg/dl)[22]. A preparation of concentrated sour cherry (*Prunus cerasus*) juice (40 g/day for 6 weeks) given to 17 women with diabetes type 2 resulted in significantly reduced HbA1c levels compared to baseline (7.9 ± 1.6 to 7.5 ± 1.2; p < 0.05) but no significant reduction in fasting glucose levels (baseline 158.3 ± 43.4 mg/dl)[23].

The possible effects of anthocyanins on diabetes as well as possible mechanisms of action have been explored in animal models. A bilberry extract added to the diet (10 g anthocyanins/kg diet) of male KK-Aγ mice (a type 2 diabetic model) for 5 weeks ameliorated hyperglycemia and increased insulin sensitivity compared to control animals. In this study, stimulation of AMP-activated protein kinase was measured in white adipose tissue, skeletal muscle and the liver. This effect was accompanied by an upregulation of glucose transporter 4 in adipose tissue and muscle, and a suppression of glucose production and lipid content in the liver[24]. A black soybean seed coat preparation, delivering a dose of 50 mg anthocyanins/kg by gavage for 30 days, was reported to increase expression of glucose transporter 4 proteins in skeletal muscle and heart tissue in streptozotocin-induced diabetic rats[25]. Purified cyanidin 3-glucoside (2 g/kg diet for 5 weeks) ameliorated hyperglycemia and increased insulin sensitivity in diabetic mice. The authors reported that cyanidin 3-glucoside upregulated glucose transporter 4 and down regulated retinol binding protein 4 expression[26].

In the present study, total and LDL cholesterol were reduced as well as the ratio of total cholesterol to HDL-cholesterol. A reduction in total and LDL-cholesterol coupled with an increase in HDL-cholesterol is considered to be a step towards prevention of cardiovascular disease. The results of this study are in line with those reported in a rat study in which açai pulp reduced total and non-HDL cholesterol in treated animals compared to controls. In this study, the animals were fed a hypercholesterolemic diet with and without 2% açaí (dry wt/wt) for 6 weeks[15]. An extract of choke berry (3 × 100 mg/day) given to 25 subjects with metabolic syndrome for 2 months significantly decreased levels of total cholesterol, LDL and triglycerides compared to baseline, while HDL levels did not change[22]. However, the previously mentioned preparation of concentrated sour cherry juice (40 g/day for 6 weeks) given to women with diabetes type 2 did not significantly alter lipid levels[23].

Treatment with açai did not affect blood pressure in this study. An effect on blood pressure was suggested by an in vitro study conducted using an extract of açai fruit (injections of 10 to 100 mg extract) added to rat mesenteric vascular bed previously contracted with norepinephrine. The study demonstrated that açai had an endothelium-dependent vasodilator effect due to effects on production of nitric oxide[27].

C-reactive protein (CRP) is one of the acute phase proteins that increases during systemic inflammation and measuring CRP levels in the blood is useful as an indicator of cardiovascular disease risk. An effect of anthocyanins on CRP in has not been established in previous clinical studies. This was also the case in a recently reported 2 month study in which an extract of choke berry (3 × 100 mg/day) was given to 25 subjects with metabolic syndrome for 2 months[22]. In accordance with previous results, this study did not demonstrate any significant change in levels of hs-CRP following administration of açai.

Exhaled nitric oxide (eNO) is an indicator of airway inflammation. There was no indication of airway inflammation in subjects in this study at baseline and no increase in eNO following the standardized meal. There is a report of an increase in eNO following a high fat meal (74 g fat; 1 g fat/kg body weight) in healthy subjects[28]. However that finding was not repeated in this study.

Açai pulp was safely consumed in this study at a dose of 100 g twice daily for one month. Experiments with mice found that doses of 3.3 to 16.7 g açaí pulp per kg body weight, given i.p. once or daily for 14 consecutive days, did not cause any genotoxic effects[29].

This was an open label, uncontrolled, pilot study designed to explore the potential effects of Sambazon^® ^Açai on risk factors for diabetes and cardiovascular disease. The limitations of this study are a lack of a blinding, a placebo control and the small sample population. This was an exploratory study, and as the results are positive, further studies placebo-controlled studies with a larger study population are warranted.

## Conclusions

In this uncontrolled pilot study, consuming 200 g per day of Sambazon^® ^Açai for 1 month reduced fasting levels of plasma glucose, insulin, and total cholesterol compared to baseline levels in a cohort of 10 overweight adults. Administration of Açai for 30 days also attenuated the post-prandial glucose response (AUC) following a standardized meal compared to the pre-treatment response. Sambazon^® ^Açai was administered safely, without adverse events. The results of this study suggest that conducting a larger placebo-controlled trial to determine the effects of acai on risk factors for chronic disease is warranted.

## Competing interests

The authors declare that they have no competing interests.

## Authors' contributions

JKU conceptualized the study and was the Principal Investigator. BBS also participated in the design of the study. BBS and VJS performed the analysis. JKU, BBS and MLB contributed to writing the manuscript. All authors have read and approved the final manuscript.
